# Facilitators and Barriers to Take Up Clinician-Collected and Self-Collected HPV Tests among Chinese Men Who Have Sex with Men

**DOI:** 10.3390/v13040705

**Published:** 2021-04-19

**Authors:** Zixin Wang, Yuan Fang, Ngai Sze Wong, Mary Ip, Xin Guo, Samuel Yeung Shan Wong

**Affiliations:** 1 Faculty of Medicine, JC School of Public Health and Primary Care, The Chinese University of Hong Kong, Hong Kong, China; candy_wong@cuhk.edu.hk (N.S.W.); mitk@cuhk.edu.hk (M.I.); 1155155162@link.cuhk.edu.hk (X.G.); 2 Department of Early Childhood Education, The Education University of Hong Kong, Hong Kong, China; lunajoef@gmail.com

**Keywords:** clinician-collected HPV tests, self-collected HPV tests, behavioral intention, facilitators and barriers, Health Belief Model, men who have sex with men

## Abstract

Regular tests for human papillomavirus (HPV) and early treatment could represent an important strategy for preventing anal cancers among men who have sex with men (MSM). This study investigated facilitators and barriers to take up clinician-collected and self-collected HPV tests among Chinese MSM. This study was based on the baseline sample of a longitudinal study promoting HPV vaccination among 350 Chinese MSM who had never received an HPV vaccination. The baseline survey was conducted from August 2019 to April 2020. The prevalence of any HPV tests uptake in lifetime was 19.1%; 4.9% had HPV infection in anus, genital, oral cavity, and other places. Among the participants, 20% and 76.8% intended to take up self-financed and free clinician-collected HPV tests, and 76.8% intended to use free self-collected HPV tests. After adjusting for significant background characteristics, perceived risk of HPV infection, and perceived benefits, barriers, cue to action, and self-efficacy related to HPV tests in general and/or specific to self-collected HPV tests were associated with behavioral intention to take up free clinician-collected and/or self-collected HPV tests. Less than 20% of Chinese MSM reported HPV tests uptake. Modifying perceptions related to HPV tests may be useful to increase HPV tests coverage in this group.

## 1. Introduction

Men who have sex with men (MSM) are at high risk of human papillomavirus (HPV) infection [1]. Meta-analyses reported a high overall prevalence of genital HPV infection among MSM both internationally (63.9% in HIV-negative MSM and 92.6% in HIV-infected MSM) [2] and in China (66.3% among MSM in general) [3]. The prevalence of HPV infection among MSM was much higher than that of the male general population [4,5,6]. In addition to high prevalence of genital warts [7], MSM’s anal cancer risk is 32–52 times higher than that of the general population [8]. Over the past 20–30 years, the incidence of squamous cell anal cancers, 91% of which are caused by persistent infection of HPV types 16 and/or 18, has increased by about 96% in men in the United States [9,10,11]. The HPV-related cancer risk was the highest among HIV-infected MSM, which accounted for 9.9% of Chinese MSM in 2016 [12].

HPV tests for MSM is most commonly done through anal cytology, which identifies patients with cellular changes in the epithelial cells that line the anal canal, and refers patients with abnormal results to high-resolution anoscopy and subsequent treatment of biopsy-confirmed anal intraepithelial neoplasia (AIN) [13]. HPV DNA testing was also used by previous studies to identity the presence of penile or oral HPV infection among MSM [14,15]. Regular tests for HPV and early treatment of dysplastic lesions could represent an important strategy for preventing anal cancers among MSM [10]. Mathematic models suggested that regular tests for HPV and anal cancer would increase quality-adjusted life expectancy of MSM and is considered as cost-effective [16,17,18]. Some international health authorities recommend HPV tests through anal cytology every 2–3 years for HIV-negative MSM and every 2 years for HIV-positive individuals [19,20]. Some practitioners have adopted HPV tests as a standard intervention for MSM [21]. In Hong Kong, there was no governmental HPV or anal cancer screening program targeting MSM. Free HPV tests are available at public hospitals or social hygiene clinics for men [22]. Some non-governmental organizations (NGOs) are also running projects providing free or chargeable HPV testing services to MSM [23]. MSM can also seek chargeable HPV testing services at private hospitals/clinics.

In the literature, the prevalence of HPV tests uptake was low among MSM, ranging from 11–14.3% among MSM in general [24,25] to 40–60% among HIV-positive MSM [26,27,28]. To our knowledge, at least five quantitative studies investigated factors associated with HPV tests uptake among MSM [24,25,27,28,29]. These factors included those related to socio-demographics (e.g., age and ethnicity), sexual behaviors (e.g., number of sex partners), presence of physical symptoms or with high-grade intraepithelial lesions, knowledge of testing procedures, and being recommended by healthcare providers to receive such tests [24,25,27,28,29]. HIV-positive MSM were more likely to receive HPV tests [25]. Perceptions had significant influences on HIV and other sexually transmitted infections (STI) screening behaviors among MSM, and they are modifiable through interventions [30,31,32]. Qualitative studies suggested that perceived stigma originated from service providers related to their sexual orientation or HIV sero-status; barriers for MSM to receive HPV tests included embarrassment and discomfort, fear of positive results, and perceived low risk of anal cancers [26,33,34]. These factors were considered by this study. In this study, we used the Health Belief Model (HBM) as the framework to guide our examination of perceptions related to HPV tests [35]. It postulates that perceived susceptibility and perceived severity of HPV infection, together with perceived benefit, perceived barrier, cue to action, and self-efficacy related to HPV tests, are determinants of the behavior. The HBM is commonly used in explaining and promoting HIV and other STI screening behaviors among MSM [30,31,32].

Self-collected HPV tests refer to collecting samples by users instead of clinicians at home or other private places for HPV testing. Users can send the collected sample to designated clinics, who will examine the sample and inform them of the results [13]. Self-collected HPV tests have been shown to have similar accuracy as clinician-collected tests [36]. Two studies showed high acceptance of self-collected HPV tests among MSM (67–78%) [26,37]. MSM perceived self-collected HPV tests to be convenient and would lower the embarrassment and fear of being stigmatized [26,37]. Self-collected HPV tests have the potential of increasing coverage of HPV tests among MSM.

To our knowledge, there was a dearth of studies investigating HPV testing behaviors or behavioral intention to receive clinician-collected or self-collected HPV tests among Chinese MSM. To address these gaps, this study investigated HPV testing behaviors and behavioral intention to take up free clinician-collected HPV tests and free self-collected HPV tests among a sample of Chinese MSM in Hong Kong. Potential associated factors were also investigated, which included socio-demographics, behavioral characteristics of the participants, perceptions related to HPV tests in general, and perceptions specific to self-collected HPV tests.

## 2. Methods

### 2.1. Study Design

Questions asked by this study was parts of the baseline survey of a longitudinal study promoting HPV vaccination among Chinese MSM, which was conducted from August 2019 to April 2020.

### 2.2. Participants

Inclusion criteria are as follows: (1) Hong Kong Chinese-speaking males aged at least 18 years old, (2) self-reported having had oral or anal intercourse with at least one man in the last six months, and (3) willing to complete a follow-up telephone survey twelve months after the baseline survey. Those who had received HPV vaccination were excluded.

### 2.3. Sampling and Data Collection

Participants were recruited through multiple sources. Trained and experienced staff of a collaborative NGO providing HIV-related services worked as fieldworkers for recruitment. A recent mapping exercise was conducted by the government and identified 12 gay bars and 16 gay saunas in Hong Kong. Upon approval of the owners, field workers approach prospective MSM participants in these venues at different time slots during weekdays and weekends. They briefed prospective participants about study details and gave them an information sheet. The research team also conducted online outreach by posting information about the study periodically as discussion topics on two gay websites with highest traffic in Hong Kong. If prospective participants are interested in this study, they can contact the interviewers through private messaging or other means (e.g., WhatsApp, telephone, email, etc.). Recruitment is supplemented by referrals made by peers and NGOs. Participants were guaranteed anonymity during the study, and they had the right to end participation in the study at any time. Their refusal or withdrawal from the study will not affect their access to any future services. Verbal instead of written informed consent was obtained due to maintaining anonymity, but the fieldworkers signed a form pledging that the participants had been fully informed about the study. Multiple forms of contact information were obtained in order to make an appointment to conduct the telephone interview. Upon completion of each survey, an HK $25 (US $3.2) supermarket or café coupon was mailed to participants as compensation for their time. Ethics approval was obtained from the Survey and Behavioral Research Ethics Committee of the Chinese University of Hong Kong (ref# KPF18HLF22).

### 2.4. Measurements

#### 2.4.1. Development of the Questionnaire

A panel that consisted of public health researchers, epidemiologists, health psychologists, physicians, and NGO workers was formed to develop the questionnaire. The questionnaire was tested among five MSM. Based on their feedback, the questionnaire was finalized by the panel.

#### 2.4.2. Background Characteristics of the Participants

Information collected included socio-demographics (age, relationship status, highest education level attained, employment status, and monthly personal income), sexual orientation, lifestyles (smoking and drinking), HIV and STI status, utilization of HIV or STI prevention services, sexual behaviors with regular and non-regular male sex partners, male sex workers, and female sex partners in the last six months. A regular male sex partner (RP) was defined as a stable boyfriend, while a non-regular male sex partner (NRP) was defined as a man who was neither a RP nor a male sex worker. Participants also reported sexualized drug use (SDU) in the last six months. SDU is defined as the use of any of the following psychoactive substance before or during sexual intercourse: ketamine, methamphetamine, cocaine, cannabis, ecstasy, Dormicum/Halcion/Erimin 5/non-prescription hypnotic drugs, heroin, cough suppressant (not for curing cough), gamma-hydroxybutyric acid (GHB)/gamma-butyrolactone (GBL), 5-methocy-N, N-diisopropyltryptamine (Foxy), or mephedrone [38,39].

#### 2.4.3. Behaviors and Behavioral Intention Related to HPV Tests

Participants were asked whether they had taken up any types of HPV tests in the lifetime and in the past year. Details related to the most recent episode of HPV test (e.g., location, cost, and testing results) were collected among MSM who had ever used HPV tests. Participants were briefed with the following statement: “Doctors can use an HPV test to identify HPV infection. Doctors can use a swab to collect samples from anus, genital, or oral cavity and examine whether you are infected with some types of HPV.” Then, they were asked about the likelihood of taking up free clinician-collected HPV tests (response categories: 1 = very unlikely, 2 = unlikely, 3 = neutral, 4 = likely, and 5 = very likely). The chance of taking up self-collected free HPV tests was also measured after participants were briefed about the following: “You can also collect the sample by yourself at home or other private places for HPV testing. The commercial package includes a swab (such as a long and thin Q-tip), you can insert the swab about 1.5–2 inches into your anus to collect the sample. You can also use the swab to collect the sample from the surface of anus or penis. You may then put the swab in the specimen bag within the commercial package and send it to designated clinics, who will examine the sample and inform you of the results” (response categories: 1 = very unlikely, 2 = unlikely, 3 = neutral, 4 = likely, and 5 = very likely). Behavioral intention to use clinician-collected and self-collected HPV tests was defined as the responses likely or very likely. Such definition has been used in previous studies [40].

#### 2.4.4. Perceptions Related to HPV tests in general Based on the HBM

Three scales derived from the HBM were constructed. Perceived severity of HPV infection was measured by four items (e.g., “HPV infection would increase risk of HIV infection”). The Perceived Severity Scale was formed by summing up individual scores (from 1 = strongly disagree to 5 = strongly agree). Higher score on the scale indicated perceived consequences of HPV infection to be more severe. The Cronbach’s α of the Perceived Severity Scale was 0.72; one factor was identified by exploratory factor analysis, explaining 54.9% of total variance.

Two items (with response options ranging from 1 = strongly disagree to 5 = strongly agree) were used to measure the perceived benefit of HPV tests and perceived barriers to receive HPV tests. The Perceived Benefit Scale and Perceived Barriers Scale were formed by summing up individual item scores. The Cronbach’s α of these two scales was 0.71 and 0.75, respectively. Single factors were identified by exploratory factor analysis, explaining 77.9% and 57.4% of total variance.

One item measured perceived susceptibility to HPV (i.e., “How high is your chance of contracting HPV in lifetime”) with response options ranging from 1 = very low to 5 = very high. Two other single items assessed cue to action (i.e., “People who are important to you suggest that you take up HPV tests”), and perceived self-efficacy (i.e., “It is easy for you to take up HPV tests in the next year if you want to”) (response categories: from 1 = strongly disagree to 5 = strongly agree).

#### 2.4.5. Perceptions Specific to Self-Collected HPV Tests

Three items measured perceived benefits specific to self-collected HPV tests (e.g., “performing self-collected HPV tests is easy for you”). The Perceived Benefit Specific to Self-collected HPV Tests Scale was constructed by summing up individual item scores (1 = strongly disagree to 5 = strongly agree). Higher score indicated perceived higher benefits of self-collected HPV tests. The Cronbach’s α of the Perceived Severity Scale was 0.67; one factor was identified by exploratory factor analysis, explaining 65.6% of total variance. Three other single items assessed perceived barrier (i.e., “You are concerned about the accuracy of self-collected HPV tests”), cue to action (i.e., “People who are important to you suggest that you take up self-collected HPV tests”), and perceived self-efficacy (i.e., “It is easy for you to take up self-collected HPV tests in the next year if you want to”) related to self-collected HPV tests (response categories: from 1 = strongly disagree to 5 = strongly agree).

### 2.5. Statistical Analysis

Using behavioral intention to use free clinician-collected HPV tests and free self-collected HPV tests as the dependent variables, and background characteristics as independent variables, crude odds ratios (OR) were obtained by using logistic regression models. After adjustment for those variables with *p* < 0.05 in the univariate analysis, associations between independent variables of interest (perceptions related to HPV tests in general and/or perceptions specific to self-collected HPV tests) and the dependent variables were then assessed by adjusted odds ratios (AOR). Each AOR was obtained by fitting a single logistic regression model, which involved one of the independent variables of interest and significant background variables. Principal component analysis with varimax rotation was used to perform exploratory factor analysis. SPSS version 21.0 was used for data analysis, with *p* values < 0.05 taken as statistically significant.

## 3. Results

### 3.1. Background Characteristics of the Participants

Among 565 prospective participants being approached, none of them had already received HPV vaccination. A total of 400 MSM were screened to be eligible, 50 refused to participate in the study for time and/or other logistical reasons, and 350 (87.5%) completed the baseline survey. About half of the participants were 18 to 30 years old (*n* = 176, 50.3%) and with a monthly personal income of HK $20,000 (US $2565) or higher (*n* = 201, 57.4%). Most of the participants were currently single (*n* = 265, 75.7%), had attained at least tertiary education (*n* = 303, 86.6%), were employed full-time (*n* = 290, 82.9%), and identified themselves as homosexuals (*n* = 319, 91.1%). Among the participants, 1.7% (*n* = 6) self-reported as living with HIV, 23.1% (*n* = 81) reported a history of other STI and 23.1% (*n* = 81) utilized HIV or STI prevention services such as receiving free condoms, peer education or pamphlet, or attending seminars or workshops. In the past six months, 83.7% (*n* = 293), 48.6% (*n* = 170), and 9 (2.6%) had had anal intercourse with RP, NRP, and male sex workers, respectively. Moreover, 50.3% (*n* = 176) and 54.6% (*n* = 191) reported CAI with men and multiple male sex partnerships, respectively. In addition, 6 (1.7%) participants had sexual intercourse with female sex partners, and 3 (0.9%) of them reported condomless sex with such partners. The prevalence of SDU was 5.7% (*n* = 20) (Table 1).

### 3.2. Behaviors and Behavioral Intention Related to HPV Tests

Among the participants, the prevalence of taking up any HPV tests in lifetime and in the past year was 19.1% (*n* = 67) and 9.7% (*n* = 34), respectively. Among 67 participants with experience of HPV tests, 25% (*n* = 17) were diagnosed as HPV infection in anus (*n* = 11), genital (*n* = 4), oral cavity (*n* = 2), and other place (*n* = 1). Their most recent episode of HPV tests were all clinician-collected HPV tests which mainly took place in NGOs (*n* = 24, 36%) followed by public hospitals (*n* = 18, 27%) and private clinics (*n* = 17, 25%). Over half of them received free HPV tests, while others spent HK $350–7000 (US $44.9–897.4) (median: HK $700 or US $89.7) for their most recent episode of HPV tests. The prevalence of behavioral intention to receive self-financed (at HK $700 or US $89.7 per episode) and free clinician-collected HPV tests in the next year was 20% (*n* = 70) and 76.8% (*n* = 269), respectively. The prevalence of behavioral intention to take up self-collected HPV tests in the next year was 76.8% (*n* = 269) (Table 2).

### 3.3. Factors Associated with Behavioral Intention to Take Up Free Clinician-Collected HPV Tests

In univariate analysis, participants who had never received HIV testing (OR: 0.23, 95%CI: 0.10, 0.55, *p* < 0.001) and had anal intercourse with RP in the past six months (OR: 0.41, 95%CI: 0.18, 0.95, *p* = 0.04) reported lower intention to use free clinician-collected HPV tests, while the history of other STI (OR: 2.92, 95%CI: 1.39, 6.15, *p* = 0.005) was positively and significantly associated with this dependent variable (Table 3).

After adjusting for these significant background characteristics, perceived benefits of HPV tests (AOR: 1.70, 95%CI: 1.40, 2.04, *p* < 0.001), belief that significant others would suggest they take up HPV tests (cue to action) (AOR: 1.71, 95%CI: 1.32, 2.22, *p* < 0.001), and perceived self-efficacy to take up HPV tests (AOR: 2.08, 95%CI: 1.55, 2.78, *p* < 0.001) were associated with higher intention to take up free clinician-collected HPV tests. Perceived barriers to take up HPV tests were negatively and significantly associated with this dependent variable (AOR: 0.82, 95%CI: 0.70, 0.92, *p* = 0.02) (Table 4).

### 3.4. Factors Associated with Behavioral Intention to Use Self-Collected HPV Tests

In univariate analysis, participants without a full-time work had higher intention to use self-collected HPV tests (OR: 2.59, 95%CI: 1.13, 5.96), while those with monthly personal income of HK $10,000–19,999 (US $1282–2564) (OR: 0.32, 95%CI: 0.11, 0.99; reference group: lower than HK $10,000 or US $1282), self-reported as HIV positive (OR: 0.13, 95%CI: 0.02, 0.72, *p* = 0.02; reference group: self-reported as HIV negative) and had never tested for HIV (OR: 0.22, 95%CI: 0.09, 0.52, *p* = 0.001; reference group: self-reported as HIV negative) reported lower intention to do so. (Table 3)

After adjusting for these significant background characteristics, perceived risk of contracting HPV in lifetime (AOR: 1.53, 95%CI: 1.15, 2.03, *p* = 0.003), and perceived benefits (AOR: 1.74, 95%CI: 1.43, 2.11, *p* < 0.001), cue to action (AOR: 1.64, 95%CI: 1.27, 2.12, *p* < 0.001), and self-efficacy (AOR: 2.12, 95%CI: 1.57, 2.86, *p* < 0.001) related to HPV tests were associated with higher intention to use self-collected HPV tests. A negative and significant association was found between perceived barriers to receive HPV tests and this dependent variable (AOR: 0.83, 95%CI: 0.70, 0.98, *p* = 0.03). Perceived benefits (AOR: 1.40, 95%CI: 1.23, 1.58, *p* < 0.001), cue to action (AOR: 1.97, 95%CI: 1.50, 2.58, *p* < 0.001) and perceived self-efficacy (AOR: 3.32, 95%CI: 2.33, 4.73, *p* < 0.001) specific to self-collected HPV tests were also significantly associated with this dependent variable. (Table 4).

## 4. Discussion

Similar to studies in Western countries (11–14.3%) [24,25], lifetime HPV tests uptake among Chinese MSM in Hong Kong was low (19.1%). Regular screening might be rare, as less than 10% of the participants reported HPV tests uptake in the past year. MSM in Hong Kong might have a high risk of HPV infection, as 25% of MSM who had ever tested for HPV received positive results. It is likely that some MSM with AIN are not identified and linked to treatment. Cost might be a serious concern for MSM to receive HPV tests, as the prevalence of behavioral intention to take up clinician-collected HPV tests varied from 20% at HK $700 per episode to about 80% of it was free of charge. Although it is currently unavailable for men in Hong Kong, self-collected HPV tests is an alternative option to increase HPV tests coverage among MSM in future, as 76.8% intended to use this option if it is provided for free. Such prevalence of behavioral intention was as high as that of free clinician-collected HPV tests in this study, and it was similar to those of studies targeting MSM in Western countries [26,37]. It is likely that some MSM who would not use clinician-collected HPV tests would prefer self-collected HPV tests. However, a meta-analysis showed that only about 50% of those with behavioral intention would translate it into related action [41]. Without effective health promotion, the prevalence of HPV tests may remain low even when free clinician-collected or self-collected HPV tests are available.

About 6% of the participants had never tested for HIV antibody. More attention should be given to this subgroup of MSM, as they reported lower intention to take up free clinician-collected HPV tests and self-collected HPV tests. Previous studies showed that many MSM who had never tested for HIV antibody engaged in high-risk behaviors and put themselves at a high risk for contracting HIV and HPV [31]. As compared to their HIV-negative counterparts, HIV-positive MSM had lower intention to take up self-collected HPV tests but a similar level of intention to use clinician-collected HPV tests. Clinician-collected HPV tests should be considered as an essential part for the care of HIV-infected men. Those who had anal intercourse with RP also reported lower intention to take up clinician-collected HPV tests. Previous studies showed a high level of trust and intimacy between MSM and their RP, which might lead MSM to underestimate the risk of HIV and STI transmission when having anal intercourse with the RP [42]. Similar to the previous studies, MSM with a history of STI had higher intention to take up clinician-collected HPV tests [24,25,27,28,29]. These MSM were more likely to consider STI as a serious threat to their health and might have a stronger need to take up HPV tests.

The findings provided some empirical insights for developing interventions promoting clinician-collected HPV tests. The HBM is a potentially useful framework to guide the development of future programs promoting clinician-collected HPV tests, as four out of its six constructs were significantly associated with the dependent variable in expected directions. A high proportion (78–89.7%) of participants perceived some benefits of HPV tests in early identification and treatment of HPV infection as well as anal cancer prevention. Those scored higher in the Perceived Benefit Scale were more likely to have behavioral intention to take up clinician-collected HPV tests. Future health promotion campaigns should strengthen such beliefs among local MSM. About 10% of the participants had concerns about being stigmatized by service providers and pain and discomfort caused by the tests. It is important to remove these concerns, as they were significantly associated with lower intention to take up clinician-collected HPV tests. Service providers should be made aware of such concerns among MSM. It may be useful to enhance their knowledge about MSM’s sub-culture and reduce stigmatization toward MSM; similar interventions were available [43]. It is expected that testimonials made by peers about positive experiences of HPV tests is useful in removing these concerns among MSM. Most of the Chinese MSM tend to have a very close circle of involved friends and a strong attachment to them [44]. As MSM is a highly marginalized community, individuals within it each may have a high level of perceived similarity with their MSM peers. Thus, it is expected that MSM find their peers’ experiences particularly valuable. Cue to action and perceived self-efficacy were both facilitators. Future programs should have significant others who are also MSM (e.g., peers, NGO workers) to give reminders to take up HPV tests as a strong cue to action. Facilitating MSM to form an action plan to take up HPV tests is a potentially useful strategy to improve perceived self-efficacy [45].

Enhancing perceived benefits, providing strong cue to action, improving perceived self-efficacy, and reducing concerns related to stigmatization and testing procedures related to HPV tests in general are also useful strategies to promote self-collected HPV tests among MSM in Hong Kong. In addition, future interventions should increase perceived risk of contracting HPV, as it was associated with higher intention to use self-collected HPV tests. Similar to HIV self-testing [46], perceived benefits related to the procedures and the confidentiality of the self-collected HPV tests (e.g., convenient, easy to use, lower stigma originated from service providers) were facilitators. Health communication messages on these benefits should be disseminated to MSM. Having significant others providing reminders and facilitating action planning to take up self-collected HPV tests are also potentially useful strategies.

This was one of the first studies looking at behaviors and behavioral intention related to HPV tests among Chinese MSM. Although it had the strengths of being theory-based, it had several limitations. First, the participants were recruited by non-probabilistic sampling in the absence of sampling frame. The results may not be representative of MSM in Hong Kong. As compared to participants of a large-scale survey involving more than 4000 MSM conducted by the Hong Kong Department of Health, our participants were younger and reported a lower prevalence of HIV infection [47]. Second, the sample was based on the baseline survey of an ongoing longitudinal study promoting HPV vaccination. The participants might have a higher motivation to adopt preventive behaviors. Third, we listed HPV vaccination uptake as an exclusion criterion. The prevalence of HPV vaccination was very low among MSM in Hong Kong. None of the participants being approached in this study had ever received HPV vaccination. Fourth, the scales were constructed for this study and were not validated by other studies. We performed exploratory factor analyses and reported satisfactory internal reliability measures. Fifth, the data were self-reported, and reporting bias existed. The prevalence of behavioral intention might have been over-reported, although anonymity should have reduced such bias. Moreover, we only obtained cross-sectional associations and could not establish causal relationships. Our outcomes investigated behavioral intention rather than actual behaviors. Behavioral intention is a strong predictor of actual behaviors.

## 5. Conclusions

Chinese MSM in Hong Kong reported a low HPV tests uptake. There is a strong need for improvement. The prevalence of behavioral intention to receive free clinician-collected HPV tests and free self-collected HPV tests was equally high. Modifying perceptions related to HPV tests in general and those specific to self-collected HPV tests might be useful to promote HPV tests in future health promotion campaigns.

## Figures and Tables

**Table 1:** Background characteristics of the participants (*n* = 350).

	*n*	%
**Socio-Demographics**		
Age group (years)		
18–24	48	13.7
25–30	128	36.6
31–40	133	38.0
>40	41	11.7
Relationship status		
Currently single	265	75.7
Married or cohabited with a man	85	24.3
Highest education level attained		
Secondary or below	47	13.4
Tertiary of above	303	86.6
Employment status		
Full-time	290	82.9
Part-time/unemployed/retired/students	60	17.1
Monthly personal income		
<HK $10,000 (US $1282)	37	10.6
HK $10,000–19,999 (US $1282–2564)	106	30.3
HK $20,000–39,999 (US $2565–5128)	134	38.3
≥HK $40,000 (US $5129)	67	19.1
Refuse to disclose	6	1.7
Sexual orientation		
Homosexual	319	91.1
Bisexual	31	8.9
**Lifestyles**		
Smoking in lifetime		
No	257	73.4
Yes	93	26.6
Drinking in the past year		
No	86	24.6
Yes	264	75.4
**History of HIV and other sexually transmitted infections (STI) and service utilization**		
Self-reported HIV sero-status		
Negative	311	88.9
Positive	6	1.7
Refuse to disclose	11	3.1
Had never tested for HIV antibody	22	6.3
History of other STIs		
No	269	76.9
Yes	81	23.1
Utilization of other HIV/STI prevention services (e.g., receiving free condoms, peer education and pamphlets, and attending seminars)		
No	269	76.9
Yes	81	23.1
**Sexual behaviors in the past six months**		
Anal intercourse with regular male sex partners		
No	57	16.3
Yes	293	83.7
Anal intercourse with non-regular male sex partners		
No	180	51.4
Yes	170	48.6
Anal intercourse with male sex workers		
No	341	97.4
Yes	9	2.6
Condomless anal intercourse with men		
No	174	49.7
Yes	176	50.3
Multiple male sex partnerships		
No	159	45.4
Yes	191	54.6
Sexual intercourse with female sex partners		
No	344	98.3
Yes	6	1.7
Condomless sex with female sex partners		
No	347	99.1
Yes	3	0.9
Sexualized drug use (use of psychoactive substances before or during sexual intercourse)		
No	330	94.3
Yes	20	5.7

**Table 2:** Variables related to human papillomavirus (HPV) tests among the participants (*n* = 350).

	*n*	%
**Behaviors and behavioral intention related to HPV tests**		
HPV tests in lifetime		
No	283	80.9
Yes	67	19.1
HPV tests in the past year		
No	316	90.3
Yes	34	9.7
Perceived chance to take up clinician-collected HPV tests in the next year if it cost HK $700 (US $89.7) per episode		
Very low	37	13.4
Low	108	30.9
Moderate	125	35.7
High	46	13.1
Very high	24	6.9
Perceived chance to take up clinician-collected HPV tests in the next year if it is provided for free		
Very low	7	2.0
Low	10	2.9
Moderate	64	18.3
High	90	25.7
Very high	179	51.1
Perceived chance to take up self-collected HPV tests in the next year if it is provided for free		
Very low	8	2.3
Low	19	5.4
Moderate	54	15.4
High	97	27.7
Very high	172	49.1
**Perceptions related to HPV tests in general based on the Health Belief Model**		
Perceived risk of contracting HPV in lifetime (perceived susceptibility), *n* (%) answered high/very high	89	25.4
Mean (SD)	3.0	(1.0)
Perceived severity of HPV infection, *n* (%) answered agree/strongly agree		
HPV infection would increase risk of HIV acquisition	106	30.3
HPV infection would cause penile or anal cancers	127	36.3
Genital warts would have severe harms on your health	182	52.0
Penile or anal cancers would have severe harms on your health	263	75.1
Perceived Severity Scale ^a^, mean (SD)	13.4	(3.1)
Perceived benefits of HPV tests, *n* (%) answered agree/strongly agree		
HPV tests could detect HPV infection earlier, so as to have better treatment outcomes	314	89.7
HPV tests could prevent cancers caused by HPV infection	273	78.0
Perceived Benefit Scale ^b^, mean (SD)	8.5	(1.5)
Perceived barriers to received HPV tests, *n* (%) agree/strongly agree		
Others would think you are having high risk sexual behaviors	57	16.3
HPV tests would cause pain and discomfort	38	10.9
Perceived Barriers Scale ^c^, mean (SD)	4.9	(1.7)
People who are important to you suggest that you take up HPV tests (cue to action), *n* (%) agree/strongly agree	148	42.3
Mean (SD)	3.3	(1.1)
It is easy for you to take up HPV tests in the next year if you want to (perceived self-efficacy), *n* (%) agree/strongly agree	189	54.0
Mean (SD)	3.6	(1.0)
**Perceptions specific to self-collected HPV tests**		
Perceived benefits specific to self-collected HPV tests, *n* (%) agree/strongly agree		
Performing self-collected HPV tests is easy for you	209	59.7
Performing self-collected HPV tests is very convenient	214	61.1
Using self-collected HPV tests can avoid being stigmatized by testing service providers	171	48.9
Perceived Benefits Specific To Self-collected HPV tests Scale ^d^, mean (SD)	11.0	(2.5)
You are concerned about the accuracy of self-collected HPV tests, *n* (%) agree/strongly agree	86	24.5
Mean (SD)	2.7	(1.1)
People who are important to you suggest that you take up self-collected HPV tests (cue to action), n (%) agree/strongly agree	147	42.0
Mean (SD)	3.3	(1.1)
It is easy for you to take up self-collected HPV tests in the next year if you want (perceived self-efficacy), *n* (%) agree/strongly agree	217	62.0
Mean (SD)	3.8	(1.0)

^a^ Perceived Severity Scale, four items, Cronbach’s alpha: 0.72, one factor was identified by exploratory factor analysis, explaining for 54.9% of total variance ^b^ Perceived Benefit Scale, two items, Cronbach’s alpha: 0.71, one factor was identified by exploratory factor analysis, explaining for 77.9% of total variance ^c^ Perceived Barriers Scale, two items, Cronbach’s alpha: 0.75, one factor was identified by exploratory factor analysis, explaining for 57.4% of total variance ^d^ Perceived Benefits Specific to Self-collected HPV tests Scale, three items, Cronbach’s alpha: 0.67, one factor was identified by exploratory factor analysis, explaining for 65.6% of total variance.

**Table 3:** Associations between background characteristics and behavioral intention to take up free clinician-collected and self-collected HPV tests (*n* = 350).

	Free Clinician-Collected HPV Tests		Free Self-Collected HPV Tests	
	OR (95%CI)	*p* Values	OR (95%CI)	*p* Values
**Socio-demographics**				
Age group (years)				
18–24	1.0		1.0	
25–30	0.45 (0.19. 1.11)	0.08	0.73 (0.33, 1.62)	0.44
31–40	0.50 (0.20, 1.21)	0.50	0.94 (0.42, 2.12)	0.89
>40	1.23 (0.36, 4.21)	0.74	1.09 (0.38, 3.07)	0.88
Relationship status				
Currently single	1.0		1.0	
Married or cohabited with a man	0.89 (0.50, 1.58)	0.70	0.97 (0.55, 1.73)	0.92
Highest education level attained				
Secondary or below	1.0		1.0	
Tertiary of above	1.32 (0.66, 2.64)	0.43	1.32 (0.66, 2.64)	0.43
Employment status				
Full-time	1.0		1.0	
Part-time/unemployed/retired/students	1.88 (0.89, 3.99)	0.11	**2.59 (1.13, 5.96)**	**0.03**
Monthly personal income				
<HK $10,000 (US $1282)	1.0		1.0	
HK $10,000–19,999 (US $1282–2564)	1.12 (0.45, 2.79)	0.81	**0.32 (0.11, 0.99)**	**0.048**
HK $20,000–39,999 (US $2565–5128)	0.81 (0.34, 1.95)	0.64	0.37 (0.12, 1.13)	0.08
≥HK $40,000 (US $5129)	0.96 (0.36, 2.53)	0.93	0.50 (0.15, 1.67)	0.26
Refuse to disclose	0.28 (0.05, 1.64)	0.16	0.24 (0.03, 1.77)	0.16
Sexual orientation				
Homosexual	1.0		1.0	
Bisexual	0.85 (0.37, 1.99)	0.71	0.60 (0.27, 1.34)	0.21
**Lifestyles**				
Smoking in lifetime				
No	1.0		1.0	
Yes	0.70 (0.41, 1.21)	0.20	0.82 (0.47, 1.42)	0.48
Drinking in the past year				
No	1.0		1.0	
Yes	1.19 (0.68, 2.10)	0.54	1.30 (0.74, 2.27)	0.36
**History of HIV and other sexually transmitted infections (STI) and service utilization**				
Self-reported HIV sero-status				
Negative	1.0		1.0	
Positive	0.55 (0.10, 3.06)	0.49	**0.13 (0.02, 0.72)**	**0.02**
Refuse to disclose	N.A.	N.A.	2.59 (0.33, 20.62)	0.37
Had never tested for HIV antibody	**0.23 (0.10, 0.55)**	**0.001**	**0.22 (0.09, 0.52)**	**0.001**
History of other STI				
No	1.0		1.0	
Yes	**2.92 (1.39, 6.15)**	**0.005**	1.77 (0.92, 3.40)	0.09
Utilization of other HIV/STI prevention services (e.g., receiving free condoms, peer education and pamphlets, and attending seminars)				
No	1.0		1.0	
Yes	1.59 (0.84, 3.01)	0.16	1.59 (0.84, 3.01)	0.16
**Sexual behaviors in the past six months**				
Anal intercourse with regular male sex partners				
No	1.0		1.0	
Yes	**0.41 (0.18, 0.95)**	**0.04**	0.49 (0.22, 1.09)	0.08
Anal intercourse with non-regular male sex partners				
No	1.0		1.0	
Yes	1.09 (0.66, 1.79)	0.73	0.69 (0.42, 1.14)	0.15
Anal intercourse with commercial male sex partners				
No	1.0		1.0	
Yes	0.59 (0.15, 2.43)	0.47	0.59 (0.15, 2.43)	0.47
Condomless anal intercourse with men				
No	1.0		1.0	
Yes	0.81 (0.49, 1.33)	0.41	0.86 (0.53, 1.42)	0.57
Multiple male sex partnerships				
No	1.0		1.0	
Yes	0.89 (0.54, 1.47)	0.65	0.68 (0.41, 1.14)	0.14
Sexual intercourse with female sex partners				
No	1.0		1.0	
Yes	0.29 (0.06, 1.48)	0.14	0.29 (0.06, 1.48)	0.14
Condomless sex with female sex partners				
No	1.0		1.0	
Yes	0.15 (0.01, 1.65)	0.12	0.86 (0.53, 1.42)	0.57
Sexualized drug use (use of psychoactive substances before or during sexual intercourse)				
No	1.0		1.0	
Yes	2.83 (0.64, 12.48)	0.17	2.83 (0.64, 12.48)	0.17
**History of HPV tests**				
HPV tests in lifetime				
No	1.0		1.0	
Yes	1.48 (0.75, 2.92)	0.26	0.65 (0.36, 1.17)	0.15

OR: crude odds ratios, CI: confidence interval, N.A.: not applicable. OR, 95%CI, and *p* values of variables with *p* < 0.05 were bold.

**Table 4:** Factors associated with behavioral intention to take up clinician-collected and self-collected HPV tests (*n* = 350).

	Free Clinician-Collected HPV Tests		Free Self-Collected HPV Tests	
	AOR (95%CI)	*p* Values	AOR (95%CI)	*p* Values
**Perceptions related to HPV and HPV tests based on the Health Belief Model**				
Perceived risk of contracting HPV in lifetime	1.23 (0.94, 1.62)	0.13	**1.53 (1.15, 2.03)**	**0.003**
Perceived Severity Scale	1.07 (0.98, 1.16)	0.12	1.07 (0.98, 1.17)	0.12
Perceived Benefit Scale	**1.70 (1.40, 2.04)**	**<0.001**	**1.74 (1.43, 2.11)**	**<0.001**
Perceived Barriers Scale	**0.82 (0.70, 0.97)**	**0.02**	**0.83 (0.70, 0.98)**	**0.03**
People who are important to you suggest that you take up HPV tests (cue to action)	**1.71 (1.32, 2.22)**	**<0.001**	**1.64 (1.27, 2.12)**	**<0.001**
It is easy for you to take up HPV tests in the next year if you want to (perceived self-efficacy)	**2.08 (1.55, 2.78)**	**<0.001**	**2.12 (1.57, 2.86)**	**<0.001**
**Perceptions specific to self-collected HPV tests**				
Perceived Benefits Specific To Self-collected HPV Tests Scale	N.A.	N.A.	**1.40 (1.23, 1.58)**	**<0.001**
You are concerned about the accuracy of self-collected HPV tests	N.A.	N.A.	0.88 (0.69, 1.11)	0.28
People who are important to you suggest that you take up self-collected HPV tests (cue to action)	N.A.	N.A.	**1.97 (1.50, 2.58)**	**<0.001**
It is easy for you to take up self-collected HPV tests in the next year if you want (perceived self-efficacy)	N.A.	N.A.	**3.32 (2.33, 4.73)**	**<0.001**

AOR: adjusted odds ratios, odds ratios adjusted for significant background characteristics listed in Table 3, CI: confidence interval, N.A.: not applicable. AOR, 95%CI, and *p* values of variables with *p* < 0.05 were bold.

## Data Availability

The data presented in this study are available on request from the corresponding authors. The data are not publicly available as they contain sensitive personal behaviors.
